# Supplementary figures and images for: F-Box Genes in the Wheat Genome and Expression Profiling in Wheat at Different Developmental Stages
Source: Genes (Basel). 2020 Sep 30;11(10):1154. doi: 10.3390/genes11101154 (PMC7650748; doi:10.3390/genes11101154)

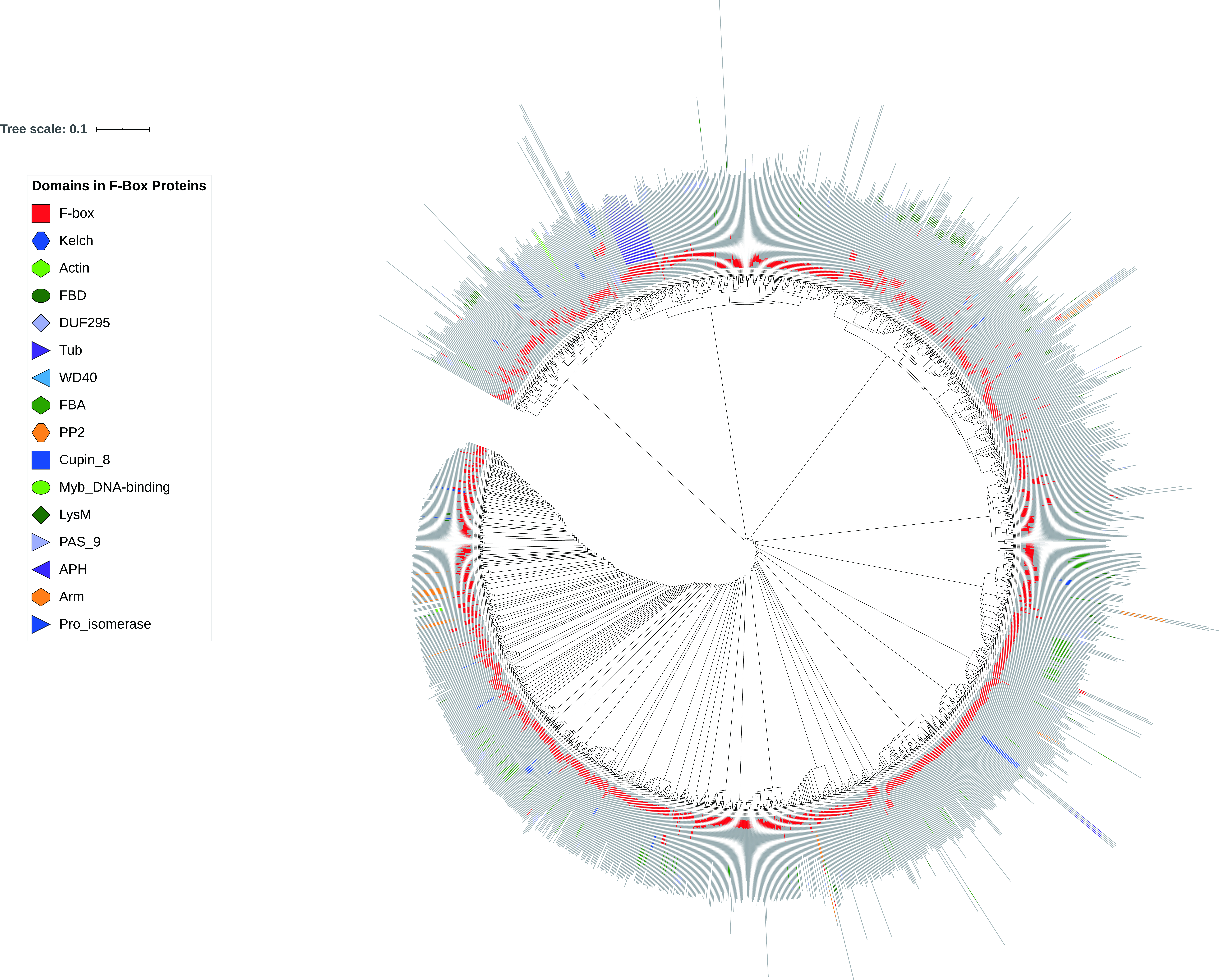

Supplement: Supplementary file 1 [file genes-11-01154-s001.zip › supple Figure 1.jpg]
